# Association between *NPPA* promoter methylation and hypertension: results from Gusu cohort and replication in an independent sample

**DOI:** 10.1186/s13148-020-00927-0

**Published:** 2020-09-03

**Authors:** Jing Li, Jinhua Zhu, Liyun Ren, Shengqi Ma, Bin Shen, Jia Yu, Rongyan Zhang, Mingzhi Zhang, Yan He, Hao Peng

**Affiliations:** 1 Department of Epidemiology, School of Public Health, Medical College of Soochow University, 199 Renai Road, Industrial Park District, Suzhou, 215123 China; 2 Department of Chronic Disease Management, Center for Disease Prevention and Control of Wujiang District, Suzhou, China; 3 Jiangsu Key Laboratory of Preventive and Translational Medicine for Geriatric Diseases, Soochow University, Suzhou, China

**Keywords:** Atrial natriuretic peptide, DNA methylation, Hypertension

## Abstract

**Background:**

Atrial natriuretic peptide (ANP), one of the main members of the natriuretic peptides system, has been associated with hypertension and related complications, but the underlying molecular mechanisms are not very clear. Here, we aimed to examine whether DNA methylation, a molecular modification to the genome, of the *natriuretic peptide A gene* (*NPPA*), the coding gene of ANP, was associated with hypertension.

**Methods:**

Peripheral blood DNA methylation of *NPPA* promoter was quantified by target bisulfite sequencing in 2498 community members (mean aged 53 years, 38% men) as a discovery sample and 1771 independent participants (mean aged 62 years, 54% men) as a replication sample. In both samples, we conducted a single CpG association analysis, followed by a gene-based association analysis, to examine the association between *NPPA* promoter methylation and hypertension, adjusting for age, sex, education level, cigarette smoking, alcohol consumption, obesity, fasting glucose, and lipids. Multiple testing was controlled by the false discovery rate approach.

**Results:**

Of the 9 CpG loci assayed, hypermethylation at 5 CpGs (CpG1, CpG3, CpG6, CpG8, and CpG9) was significantly associated with a lower odds of prevalent hypertension in the discovery sample, and one CpG methylation (CpG1 located at Chr1:11908353) was successfully replicated in the replication sample (OR = 0.82, 95%CI 0.74–0.91, *q* = 0.002) after adjusting for covariates and multiple testing. The gene-based analysis found that DNA methylation of the 9 CpGs at *NPPA* promoter as a whole was significantly associated with blood pressure and prevalent hypertension in both samples (all *P* < 0.05).

**Conclusions:**

DNA methylation levels at *NPPA* promoter were decreased in Chinese adults with hypertension. Aberrant DNA methylation of the *NPPA* gene may participate in the mechanisms of hypertension.

## Background

Hypertension, a leading modifiable risk factor for cardiovascular morbidity and mortality, affects two thirds of the population and accounts for the largest disease burden in the world [1]. Although many risk factors of hypertension have been established [2–4], decades of control efforts made through interventions against these factors did not effectively improve the prevention and management of this global epidemic which is still increasingly prevalent, indicating that some unknown mechanisms may exist. In China, nearly 245 million adults suffered from hypertension but only 15% of them were under control of blood pressure [5]. Fundamentally, blood pressure elevation reflects increased cardiac output or peripheral resistance. As an important cardiac endocrine regulatory system for the body in response to external environmental stimuli, the natriuretic peptides system plays a critical role in maintaining salt-water balance and blood pressure through natriuresis, diuresis, and vasodilation in response to cardiac output overload [6, 7]. As one of the main members of the natriuretic peptides system, atrial natriuretic peptide (ANP) may play a vital role in the development of hypertension. Indeed, the relationship between ANP and hypertension has been demonstrated by substantial evidence. For example, transgenic mice overexpressing ANP [8, 9] exhibited reduced blood pressure and mice with functional disruptions in ANP had an elevated blood pressure [10, 11] compared with their wild-type littermates. In humans, the circulating level of ANP has been associated with hypertension [12] and related vascular complications, e.g., atherosclerosis [13], heart failure [14], and stroke [15]. Polymorphisms of its coding gene—*natriuretic peptide A* (*NPPA*)—have also been associated with the susceptibility to hypertension [16–18], stroke [15, 19], myocardial infarction [20], and coronary artery disease [21, 22]. Notably, the association of rs5068 located at the *NPPA* gene with hypertension reached a genome-wide significance with a *P* value of 1 × 10^−8^ [18]. The recombinant ANP carperitide has been used in clinical practice for the management of acute decompensated heart failure [23, 24] but could cause some unfavorable effects, e.g., severe hypotension [24–26] and in-hospital death [27–29]. A better understanding of the molecular mechanisms of the blood pressure-regulating effect of ANP may help its drug development and improvement. As an interface between the fixed genome and dynamic environment, DNA methylation of gene promoters has been found to play a critical role in the regulation of transcriptional activity and gene expression [30]. Therefore, we hypothesized that aberrant methylation of the *NPPA* promoter may affect its function and subsequent ANP synthesis and excretion, thereby participating in the pathogenesis of hypertension, but this has not been studied in humans. Here, we aimed to examine the association between *NPPA* promoter methylation and hypertension in two independent samples of Chinese adults.

## Results

### Clinical characteristics of study participants

**Fig. 1:**
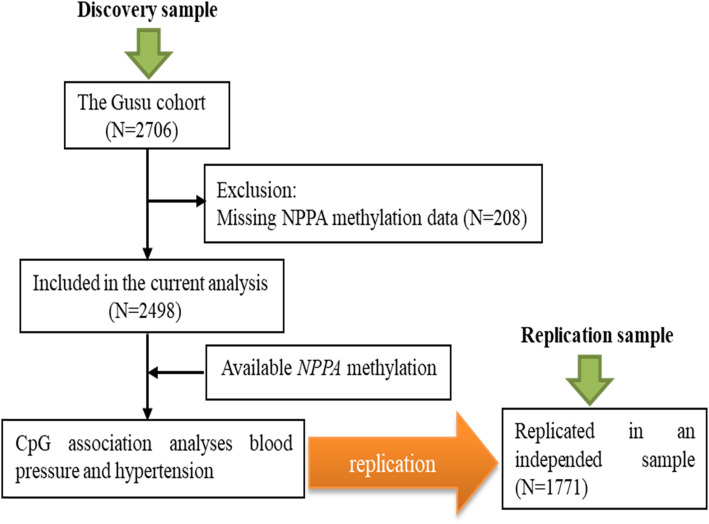
A flowchart illustrating the selection of study participants

As described in Fig. 1, a total of 2498 participants (mean aged 53 years, 38% men) in the Gusu cohort, a community-based study of cardiovascular disease and its risk factors in middle-aged and elderly Chinese adults [31], were included in the current study as the discovery sample and 1771 participants (mean aged 62 years, 54% men) in a case-control study designed to identify epigenetic markers of ischemic stroke were included as the replication sample. Of them, 1109 (44.39%) and 995 (56.18%) participants have been diagnosed with hypertension in the two samples. Their clinical characteristics were shown in Table 1. In both study samples, hypertensive participants, as expected, were more likely to be older and have more metabolic risk factors including obesity, fasting glucose, and blood lipids than those without hypertension (all *P* < 0.05).

**Table 1:** Clinical characteristics of study participants according to prevalent hypertension

Characteristics	Discovery sample			Replication sample		
	Non-HBP	HBP	*P*	non-HBP	HBP	*P*
No. of participants	1389	1109	-	776	995	-
Age, years	50.3 ± 9.3	55.7 ± 8.9	**< 0.001**	59.6 ± 12.0	63.5 ± 12.0	**< 0.001**
Sex, men *n* (%)	430 (31.0)	532 (48.0)	**< 0.001**	449 (47.0)	507 (40.1)	**0.004**
Education, high school or above *n* (%)	296 (21.3)	211 (19.0)	0.158	562 (46.9)	750 (42.5)	0.143
Current smoking, *n* (%)	281 (20.2)	301 (27.1)	**< 0.001**	324 (40.0)	318 (50.5)	**< 0.001**
Current drinking, *n* (%)	199 (14.3)	266 (24.0)	**< 0.001**	225 (42.5)	250 (47.4)	0.077
Body mass index, kg/m^2^	23.99 ± 3.30	25.78 ± 3.78	**< 0.001**	22.51 ± 3.36	24.58 ± 3.59	**< 0.001**
Fasting glucose, mmol/L	5.15 ± 1.04	5.70 ± 1.59	**< 0.001**	5.33 ± 1.82	6.31 ± 2.54	**< 0.001**
Total cholesterol, mmol/L	5.09 ± 1.52	5.38 ± 1.99	**< 0.001**	4.68 ± 1.00	5.04 ± 1.12	**< 0.001**
Triglycerides, mmol/L	1.28 ± 1.40	1.69 ± 1.77	**< 0.001**	1.52 ± 1.24	1.88 ± 4.60	**0.017**
LDL-cholesterol, mmol/L	2.92 ± 0.74	3.09 ± 0.78	**< 0.001**	3.11 ± 1.00	3.02 ± 0.96	0.053
HDL-cholesterol, mmol/L	1.55 ± 0.48	1.45 ± 0.39	**< 0.001**	1.35 ± 0.34	1.31 ± 0.39	**0.018**

All results are expressed with mean ± SD unless otherwise noted. *P* values indicate the significance level of the differences between two groups calculated by *t* test and Chi-square test as appropriate and the values in bold indicate a statistical significance level at < 0.05

*HBP* hypertension, *LDL* low-density lipoprotein, *HDL* high-density lipoprotein

### Results in the discovery sample

**Fig. 2:**
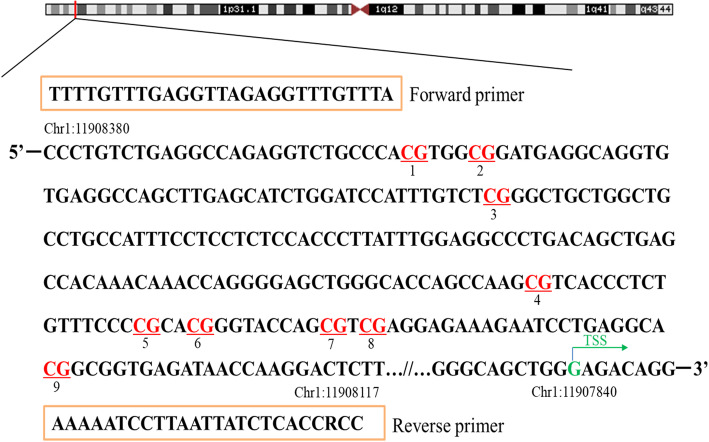
A schematic illustration of the targeted sequence and primers for targeted bisulfite sequencing. Red represents the CpG loci assayed in the *NPPA* gene promoter (− 540 to about − 277 bp from TSS). TSS, transcriptional start site

As illustrated in Fig. 2, DNA methylation levels at a total of 9 CpG loci in the promoter region of the *NPPA* gene were assayed by target bisulfite sequencing using genomic DNA isolated from peripheral blood mononuclear cells. DNA methylation levels at all of the 9 CpG loci assayed were significantly lower in participants with hypertension than those without after correction for multiple testing by adjusting for the total number of CpG loci tested using the false discovery rate (FDR) approach (all *q* < 0.05, Table 2). Of them, hypomethylation of 3 CpGs was significantly associated with either SBP or DBP, independent of conventional risk factors including age, sex, education level, cigarette smoking, alcohol consumption, body mass index (BMI), fasting glucose, lipids, and antihypertensive medications. After correction for multiple testing, hypermethylation at only 1 CpG (CpG1 located at Chr1:11908353) was still significantly associated with a lower level of SBP (*β* = − 0.96, *q* < 0.05). Hypermethylation at 4 additional CpGs (CpG3 located at Chr1:11908299, CpG6 located at Chr1:11908178, CpG8 located at Chr1:11908165, and CpG 9 located at Chr1:11908142) was also significantly associated with a lower odds of prevalent hypertension after adjusting for covariates and multiple testing (all *q* < 0.05). As expected, DNA methylation levels at the neighboring CpG sites were highly correlated (Supplementary Figure S1), suggesting a co-methylation pattern in the promoter region of the *NPPA* gene. Therefore, we examined whether DNA methylation at *NPPA* promoter, rather than a single CpG site, was associated with hypertension. The results showed that the average methylation level of all CpGs was significantly associated with a lower risk of prevalent hypertension (OR = 0.88, 95%CI 0.81–0.97, *P* = 0.008). Also, the weighted truncated product method (wTPM), an approach combines the effect of each single CpG methylation, found that DNA methylation of the 9 CpGs at the *NPPA* promoter as a whole was significantly associated with SBP, DBP, and prevalent hypertension (all *P* < 0.05, Table 3).

**Table 2:** The levels of *NPPA* promoter methylation in participants with and without hypertension

CpG loci	Genomic position, GRCh37	Relative to TSS, bp	Discovery sample				Replication sample			
			non-HBP	HBP	*P*	*q*	non-HBP	HBP	*P*	*q*
CpG1	Chr1:11908353	− 513	29.02 ± 5.13	27.96 ± 5.28	**< 0.001**	**< 0.001**	28.95 ± 5.24	27.17 ± 5.05	**< 0.001**	**< 0.001**
CpG2	Chr1:11908348	− 508	93.27 ± 2.46	93.03 ± 2.59	**0.019**	**0.025**	93.02 ± 2.40	92.92 ± 2.72	0.420	0.420
CpG3	Chr1:11908299	− 459	22.99 ± 3.84	22.64 ± 3.83	**0.024**	**0.027**	22.91 ± 4.20	22.45 ± 3.76	**0.016**	**0.049**
CpG4	Chr1:11908200	− 360	68.59 ± 6.36	67.90 ± 6.62	**0.008**	**0.013**	68.23 ± 6.74	67.67 ± 6.85	0.086	0.154
CpG5	Chr1:11908182	− 342	81.96 ± 4.81	81.32 ± 4.98	**0.001**	**0.002**	81.53 ± 4.93	81.14 ± 5.28	**0.011**	0.163
CpG6	Chr1:11908178	− 338	40.43 ± 6.05	39.60 ± 6.18	**< 0.001**	**0.002**	40.35 ± 6.18	39.12 ± 6.20	**< 0.001**	**< 0.001**
CpG7	Chr1:11908168	− 328	50.67 ± 6.34	49.81 ± 6.54	**< 0.001**	**0.002**	50.14 ± 6.39	49.71 ± 6.77	**0.017**	0.219
CpG8	Chr1:11908165	− 325	31.13 ± 6.33	30.11 ± 6.48	**< 0.001**	**< 0.001**	30.48 ± 6.41	29.82 ± 6.56	**0.033**	0.075
CpG9	Chr1:11908142	− 302	36.84 ± 7.63	36.15 ± 7.87	**0.027**	**0.027**	36.21 ± 7.75	36.63 ± 7.91	**0.026**	0.291
Average			50.55 ± 4.69	49.84 ± 4.88	**< 0.001**	-	50.20 ± 4.78	49.62 ± 5.06	**0.014**	-

*P* values indicate the significance level of the differences between two groups calculated by *t* test. *q* values indicate the significance level after correction of multiple testing by false discovery rate approach. The values in bold indicate a statistical significance level at < 0.05

**Table 3:** The associations of *NPPA* promoter methylation with blood pressure and hypertension in the discovery sample

CpG loci	Systolic blood pressure			Diastolic blood pressure			Prevalent hypertension		
	*β* (SE)^a^	*P*	*q*	*β* (SE)^a^	*P*	*q*	OR (95%CI)^b^	*P*	*q*
Single CpG association									
CpG1	− 0.96 (0.29)	**< 0.001**	**0.007**	− 0.42 (0.16)	**0.009**	0.084	0.87 (0.80–0.95)	**0.002**	**0.008**
CpG2	0.31 (0.59)	0.604	0.776	0.05 (0.33)	0.882	0.882	0.91 (0.77–1.09)	0.305	0.305
CpG3	− 0.29 (0.39)	0.454	0.681	− 0.24 (0.22)	0.276	0.414	0.88 (0.78–0.98)	**0.023**	**0.042**
CpG4	0.01 (0.23)	0.959	0.959	− 0.11 (0.13)	0.379	0.488	0.94 (0.88–1.01)	0.084	0.094
CpG5	0.06 (0.30)	0.850	0.956	− 0.08 (0.17)	0.622	0.700	0.91 (0.83–1.00)	**0.042**	0.056
CpG6	− 0.48 (0.24)	**0.047**	0.140	− 0.24 (0.14)	0.076	0.229	0.92 (0.87–0.99)	**0.022**	**0.042**
CpG7	− 0.22 (0.23)	0.347	0.625	− 0.18 (0.13)	0.166	0.299	0.93 (0.87–1.00)	**0.044**	0.056
CpG8	− 0.55 (0.23)	**0.018**	0.080	− 0.30 (0.13)	**0.022**	0.098	0.89 (0.83–0.96)	**0.001**	**0.008**
CpG9	− 0.21 (0.19)	0.268	0.602	− 0.16 (0.11)	0.137	0.299	0.94 (0.89–0.99)	**0.024**	**0.042**
Gene-based association									
Average	− 0.43 (0.31)	0.602	-	− 0.29 (0.17)	0.098	-	0.88 (0.81–0.97)	**0.008**	-
wTPM		**< 0.001**			**0.003**			**0.002**	

^a^*β* indicated the change of blood pressure in mmHg associated with per 5% increase in DNA methylation level, adjusting for age, sex, education level, cigarette smoking, alcohol consumption, body mass index, fasting glucose, low- and high-density lipoprotein cholesterol, and antihypertensive medications

^b^Odds of having hypertension associated with per 5% increase in DNA methylation level, adjusting for age, sex, education level, cigarette smoking, alcohol consumption, body mass index, fasting glucose, and low- and high-density lipoprotein cholesterol

The values in bold indicate a statistical significance level at < 0.05

### Results in the replication sample

**Fig. 3:**
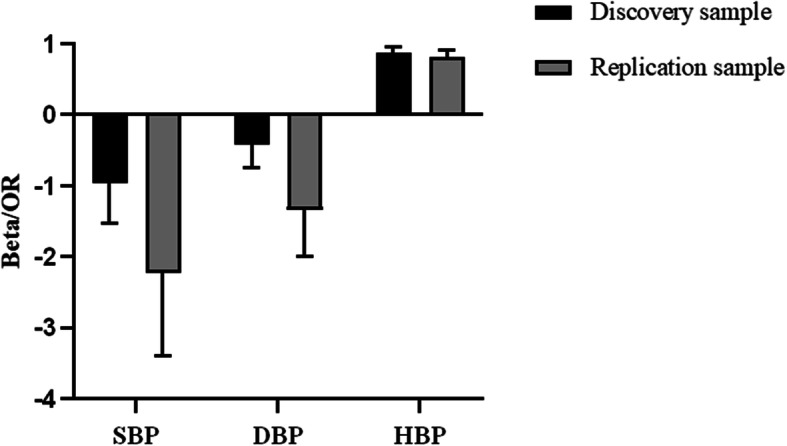
The effects of DNA methylation at CpG1 located at Chr1:11908353 on blood pressure and hypertension in the discovery and replication samples. SBP, systolic blood pressure; DBP, diastolic blood pressure; HBP, hypertension

A similar phenomenon of the association between *NPPA* promoter methylation and hypertension was observed in the replication sample. Almost all CpG methylation levels seemed to be lower in participants with hypertension than those without, but only 3 (CpG1, CpG3, CpG6) survived after the correction of multiple testing (all *q* < 0.05, Table 2). Hypomethylation of 8 CpGs including CpG1 located at Chr1:11908353 (*β* = − 2.22 for SBP and *β* = − 1.34 for DBP) were significantly associated with either SBP or DBP after adjusting for covariates and multiple testing (all *q* < 0.05, Table 4). Consistent with findings in the discovery sample, hypermethylation at CpG1 was also significantly associated with a lower odds of prevalent hypertension after adjusting for covariates and multiple testing (OR = 0.82, 95%CI 0.74–0.91, *q* = 0.002, Fig. 3). The gene-based analysis found that DNA methylation of the 9 CpGs at *NPPA* promoter as a whole was significantly associated with SBP, DBP, and prevalent hypertension (all *P* < 0.05).

**Table 4:** The associations of *NPPA* promoter methylation with blood pressure and hypertension in the replication sample

CpG loci	Systolic blood pressure			Diastolic blood pressure			Prevalent hypertension		
	*β* (SE)^a^	*P*	*q*	*β* (SE)^a^	*P*	*q*	OR (95%CI)^b^	*P*	*q*
Single CpG association									
CpG1	− 2.22 (0.60)	**< 0.001**	**0.002**	− 1.34 (0.34)	**< 0.001**	**< 0.001**	0.82 (0.74–0.91)	**< 0.001**	**0.002**
CpG2	− 2.00 (1.11)	0.072	0.093	− 1.51 (0.63)	**0.016**	**0.021**	1.05 (0.85–1.29)	0.651	0.976
CpG3	− 1.92 (0.81)	**0.018**	**0.048**	− 1.35 (0.45)	**0.003**	**0.007**	0.95 (0.84–1.09)	0.481	0.976
CpG4	− 0.87 (0.44)	**0.049**	0.073	− 0.68 (0.25)	**0.006**	**0.010**	1.00 (0.92–1.08)	0.936	0.998
CpG5	− 1.22 (0.57)	**0.033**	0.059	− 0.89 (0.32)	**0.006**	**0.010**	1.00 (0.90–1.11)	0.998	0.998
CpG6	− 1.13 (0.49)	**0.021**	**0.048**	− 0.95 (0.28)	**< 0.001**	**0.002**	0.94 (0.86–1.02)	0.158	0.475
CpG7	− 1.20 (0.45)	**0.007**	**0.033**	− 0.91 (0.25)	**< 0.001**	**0.001**	1.02 (0.95–1.11)	0.559	0.976
CpG8	− 0.59 (0.47)	0.203	0.228	− 0.54 (0.22)	**0.040**	**0.045**	1.01 (0.93–1.10)	0.780	0.998
CpG9	− 0.05 (0.39)	0.894	0.894	− 0.31 (0.22)	0.160	0.160	1.08 (1.01–1.16)	**0.018**	0.081
Gene-based association									
Average	− 1.29 (0.60)	**0.031**	-	− 1.04 (0.34)	**0.002**	-	1.05 (0.94–1.18)	0.360	-
wTPM		**< 0.001**			**< 0.001**			**0.003**	

^a^*β* indicated the change of blood pressure in mmHg associated with per 5% increase in DNA methylation level, adjusting for age, sex, education level, cigarette smoking, alcohol consumption, body mass index, fasting glucose, low- and high-density lipoprotein cholesterol, and antihypertensive medications

^b^Odds of having hypertension associated with per 5% increase in DNA methylation level, adjusting for age, sex, education level, cigarette smoking, alcohol consumption, body mass index, fasting glucose, and low- and high-density lipoprotein cholesterol

The values in bold indicate a statistical significance level at < 0.05

### Results of secondary analysis

The meta-analysis did not identify more CpG sites whose methylation levels were associated with blood pressure or hypertension (Supplementary Figure S2). Bioinformatics analysis showed that the *NPPA* gene participated in 7 KEGG pathways and 30 biological processes, most of which have been considered involved in the regulation of blood pressure (Supplementary Table S1). Gene network analysis found similar results that the *NPPA* gene may play a critical role in the pathogenesis of hypertension through varied functions, e.g., circulatory system process, regulation of blood pressure, and blood circulation (Supplementary Figure S3).

## Discussion

Leveraging two independent samples of Chinese adults, we found for the first time that DNA methylation levels of *NPPA* promoter were lower in participants with hypertension than those without. Hypermethylation at the promoter region of the *NPPA* gene was associated with a lower risk of prevalent hypertension, independent of behavior and metabolic factors. These results may suggest a potential role of aberrant *NPPA* promoter methylation in the molecular mechanisms of hypertension.

As one of the main components of the natriuretic peptides system, ANP has been widely considered a potential drug candidate for the cardiovascular system due to its biological functions of natriuresis, diuresis, vasorelaxation, and inhibition of renin and aldosterone secretion [32]. The recombinant ANP carperitide has been used in clinical practice for the management of acute decompensated heart failure [23, 24] but could cause some unfavorable effects, e.g., severe hypotension [24–26] and in-hospital death [27–29]. A better understanding of the molecular mechanisms underlying the association between ANP and hypertension may help its drug development and improvement. In line with our study, the role of its coding gene—*NPPA*—in blood pressure regulation has been suggested by previous studies [16, 17, 33–35]. For example, a meta-analysis including 4068 individuals revealed that the *NPPA* gene rs5065 polymorphism might contribute to the occurrence of hypertension [33]. A recent GWAS identified a hypertension-related SNP rs3753584 located at the *NPPA* gene which was associated with a higher circulating ANP level [35]. These findings suggest that factors regulating the function of the *NPPA* gene may conserve the potential to be the molecular mechanisms emerging to be studied that underneath the association between ANP and hypertension. As a modifiable molecular modification to the genome without changes in the genes’ sequence, DNA methylation may affect gene function and repress transcription by altering promoter DNA accessibility and blocking the binding of transcription activating proteins [36]. We, therefore, hypothesized that the *NPPA* promoter methylation may be a potential molecular modification that regulates ANP expression/excretion and participate in the pathogenesis of hypertension. Indeed, this epigenetic regulation of ANP expression has been observed by integrative analyses on DNA methylomes and transcriptomes data in humans [37]. Some hypertension-related methylation markers have been found in animals and humans. The hypomethylation of angiotensin II receptor type 1b might lead to overexpression of the gene in the adrenal glands and the development of hypertension in adult rats [38, 39]. In humans, aberrant methylation of angiotensin II receptor type 1 promoter [40] and angiotensin I converting enzyme [41] was associated with hypertension. An epigenome-wide association study including 17,010 individuals identified many CpG sites and genes in association with blood pressure, but none was related to the *NPPA* gene [42]. No study, to the best of our knowledge, has examined the role of DNA methylation at the *NPPA* gene in hypertension. Leveraging an unselected population in the Gusu cohort, we are the first to examine the association between *NPPA* promoter methylation and hypertension and provide initial evidence for the potential role of *NPPA* promoter methylation in the pathogenesis of hypertension. We found that DNA methylation level of *NPPA* promoter was more likely to be lower in participants with hypertension than those without. Hypomethylation of the *NPPA* gene was suggested to be associated with an upregulation of ANP transcription [37]. In line with our study, many studies have found that patients with hypertension had higher levels of ANP than those without and ANP levels showed a progressive elevation with the severity of hypertension [12, 43–45]. Although we did not have data on circulating ANP in our study, together with prior studies, we believed that DNA methylation of the *NPPA* gene undoubtedly participated in the molecular mechanisms of hypertension. The causality of DNA methylation of the *NPPA* gene in hypertension development is still unclear and warranted to be further studied.

In this study, we found that the contribution of an individual CpG methylation to blood pressure or hypertension was in general small (mostly < 5%), and statistically most CpG sites could not withstand multiple testing correction. Such a small effect size may not be detected by conventional statistical methods. Their combined effects may be large enough to be useful for risk prediction. Therefore, we tested the joint associations of multiple CpG methylation with blood pressure or hypertension and found significant joint associations in both sample, even though most single CpG associations did not reach a statistical significance level. Our results may unravel a molecular mechanism that *NPPA* promoter methylation may participate in the pathology of hypertension and suggest that simultaneously testing the joint effects of multiple CpG sites is a powerful approach in epigenetic analysis for complicated diseases, e.g., hypertension.

The strengths of our study include the independent replication of the association between *NPPA* promoter methylation and hypertension; comprehensive measurement and adjustment of confounding factors including lifestyles, metabolic factors, and medical history; and application of the wTPM to test the joint association of multiple CpG methylation at *NPPA* promoter with hypertension. Some limitations of our study should also be acknowledged. First, the cross-sectional study design precludes causal inference. It is still unclear whether aberrant methylation at *NPPA* promoter is a risk factor, consequence, or just an accompanying phenomenon of hypertension. Second, as in most observational studies, residual confounding is of concern. Although our results remained significant after controlling for known risk factors of hypertension and were successfully replicated in an independent sample, some unmeasured confounding effects may exist. Third, our findings were derived from Chinese adults only whose cardiovascular health profiles could be different from other populations with different ethnic backgrounds. Thus, the generalizability of our results to other ethnic populations is uncertain. Fourth, the participants in the replication sample come from a case-control study designed to identify epigenetic markers of ischemic stroke and seem to be older than those in the discovery sample. Selection bias may therefore exist in the replication sample and may overestimate the magnitude of the association between *NPPA* promoter methylation and hypertension. Fifth, we did not have data on circulating ANP levels in our study. Whether and to what extent *NPPA* promoter methylation accounts for the molecular mechanisms underlying the association between ANP and hypertension still needs more investigation, although hypomethylation of the *NPPA* gene was found to be associated with an upregulation of the transcripts of ANP [37]. Finally, given that DNA methylation is tissue- and cell-type specific, it is unclear whether or to what extent the results derived from peripheral blood could reflect methylation changes in the target organs of hypertension, e.g., cardiac and artery. However, accumulating evidence indicated that epimutations may not be limited to the affected tissue but could also be detected in peripheral blood [46–48].

## Conclusions

In conclusion, we demonstrate that hypermethylation at *NPPA* promoter is associated with a lower level of blood pressure and a lower risk of having hypertension in Chinese adults. Our results suggest a potential role of *NPPA* promoter methylation in the molecular mechanisms of hypertension.

## Methods

### Study participants

As described in Fig. 1, the current study included 2498 participants as the discovery sample and 1771 participants as the replication sample.

#### Discovery sample

The Gusu cohort is a community-based prospective longitudinal study of cardiovascular disease and its risk factors in middle-aged and elderly Chinese adults. The study design, methods, and laboratory techniques have been described previously [31]. In brief, a total of 2706 community members over 30 years were included and completed the baseline examination conducted in 2010. All participants had not been diagnosed with cardiovascular or chronic kidney disease. The protocols were approved by the Soochow University Ethics Committee. Written informed consent was obtained from all study participants. After excluding participants lacking DNA samples (*n* = 208), 2498 participants were included in the final analysis as the discovery sample.

#### Replication sample

An independent sample of 1771 participants (including 853 ischemic stroke patients and 918 age- and sex-matched healthy controls) were selected from China Antihypertensive Trial in Acute Ischemic Stroke (CATIS) [49] and Metabolic Syndrome and the Multi-metabolic Disorders Study [50], respectively, based on the availability of DNA specimens. The primary objective of this study was to identify new epigenetic markers of ischemic stroke.

### Quantification of the *NPPA* promoter methylation

In both samples, DNA methylation levels in the promoter region of the *NPPA* gene were quantified by target bisulfite sequencing as previously described [48]. using genomic DNA isolated from peripheral blood mononuclear cells. In brief, based on the genomic coordinates of the *NPPA* promoter in Genome Reference Consortium Human Build 37 (GRCh37), we carefully designed the primers to detect the maximum CpG loci within the CpG islands. The targeted sequence (Chr1: 11908117–11908380, reverse strand, relative to TSS: − 540 bp to − 277 bp) was illustrated in Fig. 2. Following primer validation, genomic DNA was bisulfite-treated using the EZ DNA Methylation-Gold Kit (Zymo Research, Inc., CA, USA) according to the manufacturer’s protocol, which converts unmethylated cytosine into uracil and leaves methylated cytosine unchanged. The treated samples were amplified, barcoded, and sequenced by Illumina Hiseq 2000 (Illumina, Inc., CA, USA) using the paired-end sequencing protocol according to the manufacturer’s guidelines. Methylation level at each CpG dinucleotides was calculated as the percentage of the methylated alleles over the sum of methylated and unmethylated alleles. For quality control, the samples with bisulfite conversion rate < 98% and the cytosine sites with average coverage less than 20× were filtered out. DNA methylation levels were finally quantified at 9 CpG loci in the *NPPA* promoter.

### Measurement of blood pressure

Blood pressure was measured three times by trained staff using a standard mercury sphygmomanometer and a cuff of appropriate size according to a standard protocol [51], after the participants had been resting for at least 5 min in a relaxed, sitting position. The first and fifth Korotkoff sounds were recorded as systolic blood pressure (SBP) and diastolic blood pressure (DBP), respectively. The mean of the three measurements was used in statistical analyses. According to the Chinese guidelines for the management of hypertension, participants with an SBP ≥ 140 mmHg and/or a DBP ≥ 90 mmHg or under antihypertensive treatment in the last 2 weeks were diagnosed with hypertension [52].

### Assessment of risk factors of hypertension

Demographic data including age, sex, and education level were obtained by questionnaires administered by trained staff. Cigarette smoking was classified as current smoking or not. Current smoking was defined as having smoked at least 100 cigarettes in the entire life, having smoked cigarettes regularly, and smoking currently. Alcohol consumption was classified as current drinkers or not. Current drinkers were those who had consumed any alcohol during the past year. Bodyweight (kg) and height (cm) were measured when participants wore light clothes and no shoes by trained staff. BMI was calculated by dividing weight in kilograms by the square of height in meters (kg/m^2^). Fasting glucose, blood lipids including total cholesterol, triglycerides, high-density lipoprotein cholesterol (HDL-C), and low-density lipoprotein cholesterol (LDL-C) were measured by standard laboratory methods [31].

### Statistical analysis

Baseline characteristics and *NPPA* promoter methylation levels were presented and compared between participants with and without hypertension using Student’s *t* test and Chi-square test as appropriate. We examined and replicated the cross-sectional association between DNA methylation levels of 9 CpG sites in the *NPPA* promoter region and prevalent hypertension in two independent samples using identical regression models. Multiple testing was controlled by adjusting for the total number of CpG loci tested using the FDR approach, and an FDR-adjusted *P* value (i.e., *q* value) of less than 0.05 was considered statistically significant. All analyses were conducted using R version 3.6.1.

### Single CpG association analysis

To examine the association between each single CpG methylation and blood pressure, we constructed a linear regression model in which blood pressure (SBP and DBP, respectively) was the dependent variable and DNA methylation at each CpG locus was the independent variable, adjusting for age, sex, education level, cigarette smoking, alcohol consumption, BMI, fasting glucose, LDL-C, HDL-C, and antihypertensive medications (y/n). To facilitate data interpretation, we similarly constructed a logistic regression model with prevalent hypertension (y/n) as the dependent variable to further examine the association between *NPPA* promoter methylation and odds of hypertension, adjusting for age, sex, education level, cigarette smoking, alcohol consumption, BMI, fasting glucose, LDL-C, and HDL-C.

### Gene-based association analysis

To examine whether DNA methylation of multiple CpG sites was jointly associated with hypertension, we treated the average methylation level of multiple CpG sites as a substitute for the methylation level of the targeted region and similarly examined its associations with blood pressure and prevalent hypertension. We also employed the wTPM as previously described [53], based on the results of a single CpG association analysis. This method combines *P* values of all CpGs that reaches a preselected threshold (e.g., raw *P* < 0.1 in this study). The regression coefficient of each individual CpG methylation was included as weights in the wTPM statistic.

### Secondary analysis

To identify more potential CpG sites, we conducted a meta-analysis using the summary data from the discovery and replication samples. The random-effects model was applied to provide more conservative estimated effects. Gene Ontology (GO) function and Kyoto Encyclopedia of Genes and Genomes (KEGG) pathway enrichment analyses were conducted for the *NPPA* gene by DAVID [54]. The regulatory network involving the *NPPA* gene was also constructed by GeneMANIA (http://genemania.org/).

## Supplementary information

**Additional file 1: Supplementary Table S1.** Biologic processes for the *NPPA* gene identified by Gene Ontology (GO). **Supplementary Figure S1.** The Pearson correlation matrix among DNA methylation levels of all CpG loci assayed in *NPPA* promoter in the discovery sample. **Supplementary Figure S2.** Results of the meta-analysis. **Supplementary Figure S3.** Gene net-work involving the *NPPA* gene.

## Data Availability

The datasets used during the current study are available from the corresponding author on a reasonable request.

## Abbreviations

ANP: Atrial natriuretic peptide
*NPPA*: Natriuretic peptide A
GRCh37: Genome Reference Consortium Human Build 37
SBP: Systolic blood pressure
DBP: Diastolic blood pressure
BMI: Body mass index
HDL-C: High-density lipoprotein cholesterol
LDL-C: Low-density lipoprotein cholesterol
FDR: False discovery rate
wTPM: Weighted truncated product method
GO: Gene Ontology
KEGG: Kyoto Encyclopedia of Genes and Genomes
