# Supplementary material for: Association between NPPA promoter methylation and hypertension: results from Gusu cohort and replication in an independent sample
Source: Clin Epigenetics. 2020 Sep 3;12:133. doi: 10.1186/s13148-020-00927-0 (PMC7469321; doi:10.1186/s13148-020-00927-0)
Supplement: Supplementary file 1 — Additional file 1: Supplementary Table S1. Biologic processes for the NPPA gene identified by Gene Ontology (GO). Supplementary Figure S1. The Pearson correlation matrix among DNA methylation levels of all CpG loci assayed in NPPA promoter in the discovery sample. Supplementary Figure S2. Results of the meta-analysis. Supplementary Figure S3. Gene net-work involving the NPPA gene. [file 13148_2020_927_MOESM1_ESM.docx]

**Supplementary data**

| **Supplementary** **Table S1**. Biologic processes for the *NPPA* gene identified by Gene Ontology (GO) | | | |
| --- | --- | --- | --- |
| GO ID | Qualified GO term | Evidence | PubMed IDs |
| GO:0001666 | response to hypoxia | IEA |  |
| GO:0003085 | negative regulation of systemic arterial blood pressure | IBA | 21873635 |
| GO:0006182 | cGMP biosynthetic process | IDA | 1672777 |
| GO:0006367 | transcription initiation from RNA polymerase II promoter | TAS |  |
| GO:0006457 | protein folding | IDA | 16870210 |
| GO:0007165 | signal transduction | IEA |  |
| GO:0007168 | receptor guanylyl cyclase signaling pathway | IBA, IDA | 1672777 |
| GO:0007218 | neuropeptide signaling pathway | IDA | 19646991 |
| GO:0007565 | female pregnancy | ISS, IEA |  |
| GO:0008217 | regulation of blood pressure | IEA, IDA | 6230082 |
| GO:0010460 | positive regulation of heart rate | IMP | 19646991 |
| GO:0010753 | positive regulation of cGMP-mediated signaling | IEA |  |
| GO:0014898 | cardiac muscle hypertrophy in response to stress | IEA |  |
| GO:0019934 | cGMP-mediated signaling | IBA, IEA | 21873635 |
| GO:0030308 | negative regulation of cell growth | IEA |  |
| GO:0032868 | response to insulin | IEA |  |
| GO:0035994 | response to muscle stretch | TAS | 15276478 |
| GO:0043508 | negative regulation of JUN kinase activity | IDA | 22399583 |
| GO:0044267 | cellular protein metabolic process | TAS |  |
| GO:0060372 | regulation of atrial cardiac muscle cell membrane repolarization | IMP | 19646991 |
| GO:0060452 | positive regulation of cardiac muscle contraction | IMP | 19646991 |
| GO:0061049 | cell growth involved in cardiac muscle cell development | IEA |  |
| GO:0071260 | cellular response to mechanical stimulus | IEA |  |
| GO:1901841 | regulation of high voltage-gated calcium channel activity | IEA, ISS | 8301666 |
| GO:1902261 | positive regulation of delayed rectifier potassium channel activity | IDA, IEA | 8301666 |
| GO:1902514 | regulation of calcium ion transmembrane transport via high voltage-gated calcium channel | ISS, IEA | 8301666 |
| GO:1903595 | positive regulation of histamine secretion by mast cell | IEA |  |
| GO:1903766 | positive regulation of potassium ion export across plasma membrane | IEA, IDA | 8301666 |
| GO:1903779 | regulation of cardiac conduction | TAS |  |
| GO:1903815 | negative regulation of collecting lymphatic vessel constriction | IEA |  |
| IMP: Inferred from Mutant Phenotype; ISS: Inferred from Sequence or Structural Similarity; IEA: Inferred from Electronic Annotation; TAS: traceable Author Statement; IDA: Inferred From Direct Assay; IBA: Inferred from Biological aspect of Ancestor. | | | |


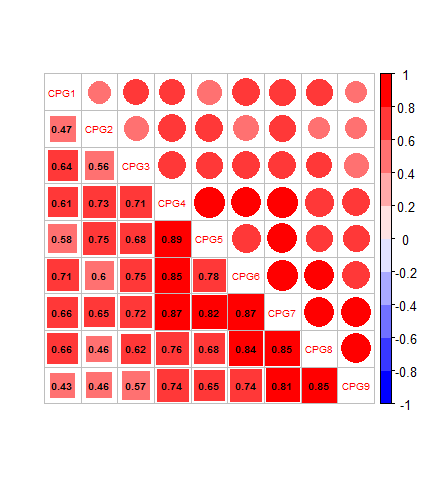


**Supplementary Figure S1.** The Pearson correlation matrix among DNA methylation levels of all CpG loci assayed in *NPPA* promoter in the discovery sample.


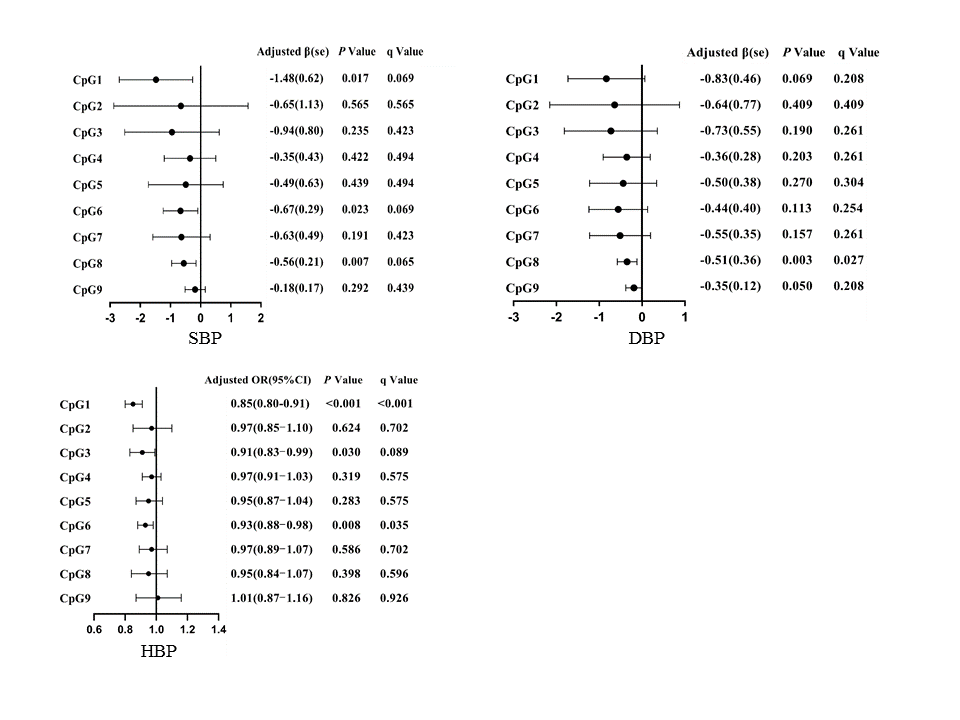


**Supplementary Figure S2.** Results of the meta-analysis.

**Supplementary Figure S3.** Gene net-work involving
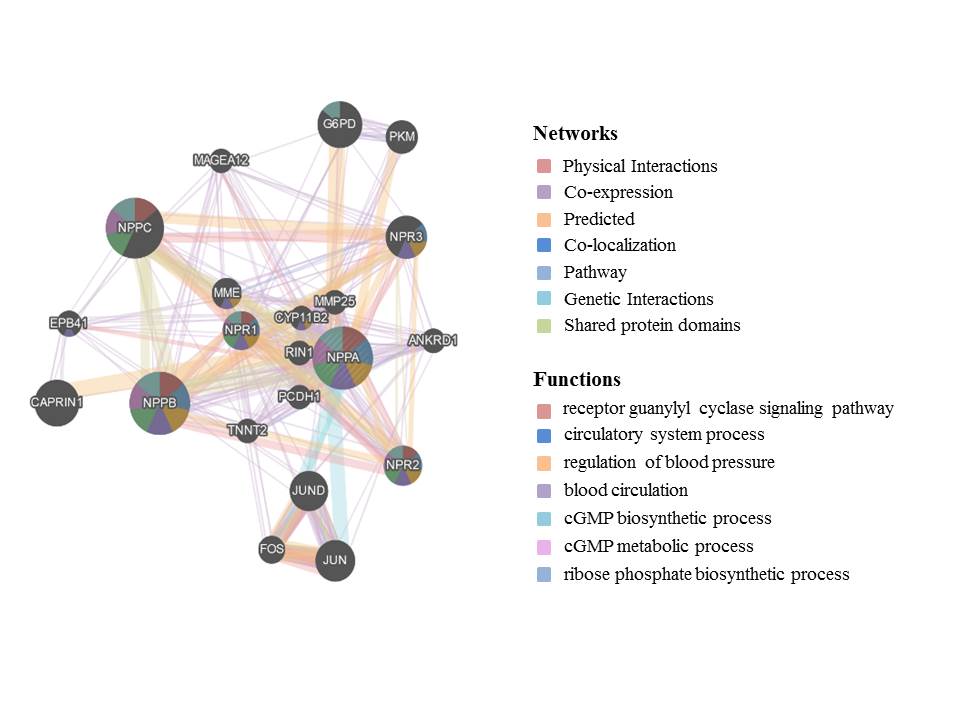
the *NPPA* gene
